# Sulforaphane Inhibits Prostaglandin E2 Synthesis by Suppressing Microsomal Prostaglandin E Synthase 1

**DOI:** 10.1371/journal.pone.0049744

**Published:** 2012-11-16

**Authors:** Jiping Zhou, Denise G. Joplin, Janet V. Cross, Dennis J. Templeton

**Affiliations:** Department of Pathology, University of Virginia School of Medicine, Charlottesville, Virginia, United States of America; National University of Ireland Galway, Ireland

## Abstract

Sulforaphane (SFN) is a dietary cancer preventive with incompletely characterized mechanism(s) of cancer prevention. Since prostaglandin E2 (PGE2) promotes cancer progression, we hypothesized that SFN may block PGE2 synthesis in cancer cells. We found that SFN indeed blocked PGE2 production in human A549 cancer cells not by inhibiting COX-2, but rather by suppressing the expression of microsomal prostaglandin E synthase (mPGES-1), the enzyme that directly synthesizes PGE2. We identified the Hypoxia Inducible Factor 1 alpha (HIF-1α) as the target of SFN-mediated mPGES-1 suppression. SFN suppressed HIF-1α protein expression and the presence of HIF-1α at the mPGES-1 promoter, resulting in reduced transcription of mPGES-1. Finally, SFN also reduced expression of mPGES-1 and PGE2 production in A549 xenograft tumors in mice. Together, these results point to the HIF-1α, mPGES-1 and PGE2 axis as a potential mediator of the anti-cancer effects of SFN, and illustrate the potential of SFN for therapeutic control of cancer and inflammation. Harmful side effects in patients taking agents that target the more upstream COX-2 enzyme render the downstream target mPGES-1 a significant target for anti-inflammatory therapy. Thus, SFN could prove to be an important therapeutic approach to both cancer and inflammation.

## Introduction

PGE2 is one of the most abundant prostaglandins in the human body, and has been implicated in numerous physiological and pathological processes, including immune responses and cancer [1]. Diverse pathological and physiological stimuli can initiate prostaglandin synthesis by first activating phospholipase A2 (PLA2), that liberates arachidonic acid (AA) from membrane phospholipids into the cytoplasm [2]. This AA is then converted into prostaglandin G2 (PGG2) and prostaglandin H2 (PGH2) by cyclooxygenase (COX). There are two isoforms of COX, designated as COX-1 and COX-2. COX-1 is constitutively expressed in most cell types and has been implicated in a number of homeostatic processes including stomach acidity, endometrial cycling and renal function [3]. In contrast, COX-2 expression is inducible, and is highly upregulated in response to infection, atherosclerosis and cancers [4], [5].

Three distinct enzymes participate in generating PGE2 from PGH2 and are designated cytosolic PGE synthase (cPGES), and microsomal PGE synthase -1 and -2 (mPGES-1 and mPGES-2) [1]. cPGES and mPGES-2 are constitutively expressed while mPGES-1 expression is inducible by inflammation [6].

The nonsteroidal anti-inflammatory drugs (NSAIDs), such as aspirin and ibuprofen, reduce PGE2 biosynthesis by inhibiting both COX-1 and COX-2, and thereby suppress inflammation, fever, and pain [7]. However, long-term use of these drugs can cause life threatening side effects, mainly gastrointestinal injury and renal pathology [8]. COX-2 specific inhibitors were designed to minimize these side effects, but recent clinical studies indicated small but significantly increased risks for cardiovascular events such as sudden myocardial infarction and thrombosis due to imbalance in the levels of PGI2 and TXA2 [9]. This suggests a need for effective and safe alternative approaches to reduce PGE2 levels. As a consequence, the focus of research has shifted to efforts to devise inhibitors for enzymes downstream of COX-2, such as mPGES-1, as potential anti-inflammatory therapies.

Isothiocyanates (chemical structure R-N = C = S) show chemopreventive activity in several models of cancer, including colon, lung, breast, stomach and prostate cancers [10]–[12]. Sulforaphane (SFN, 1-isothicyanato-4-methylsulfinyl-butane) is the major isothiocyanate present in broccoli. SFN is a potent anti-cancer agent that may function in several ways including: 1) induction of phase II detoxification enzymes and antioxidant proteins through the activation of antioxidant response element (ARE)-mediated transcriptional activity [13], 2) inhibition of cytochrome P450 enzymes [14], 3) induction of apoptosis [15]–[19], 4) suppression of cell cycle progression [20], [21], 5) inhibition of angiogenesis [22], [23] and 6) anti-inflammatory activities [24], [25]. While these activities are clear in both cell culture models and in vivo, the mechanisms by which SFN carries out these effects are not yet fully understood. These varied activities suggest that SFN may exhibit anti-cancer benefits at several stages of tumor development, including tumor initiation, promotion, progression, angiogenesis and metastasis.

One transcriptional regulator of mPGES-1 is the hypoxia-inducible factor HIF-1, that has been implicated in regulating gene expression patterns that contribute to apoptosis, angiogenesis, invasion and metastasis [26], [27]. HIF-1 is composed of two subunits, HIF-1α and HIF-1β. The HIF-1α protein level in cells is highly regulated. Under normoxic conditions, HIF-1α is constitutively expressed but rapidly hydroxylated by prolyl hydroxylase-domain oxygenases (PHD enzymes), targeting HIF-1α for degradation by an ubiquitin-proteasome pathway [28]. The synthesis of HIF-1α protein is also strictly regulated. HIF-1α accumulates during hypoxia and some inflammatory conditions. Once stabilized, HIF-1α translocates into the nucleus, dimerizes with HIF-1β, and activates the transcription of target genes such as Vascular Endothelial Growth Factor (VEGF) and Nitric Oxide Synthase 2 (NOS2), which are highly related to tumor growth and survival [27]. Multiple studies have reported significant correlations between HIF-1a accumulation and tumor progression in several types of human cancer [29], [30].

It is becoming clear that cancers are dependent upon a supportive inflammatory microenvironment, and that the cytokine IL1β contributes to this microenvironment [31]. The mechanism through which IL1β contributes to tumor formation is at least partly through activation of HIF1α by IL1β [32], [33]. Further, the development of one significant cell type within the inflammatory environment, myeloid derived suppressor cells, is enhanced by production of prostaglandin E2 by tumor cells [34]. From these observations we developed the hypothesis that SFN may reduce the expression of PGE2 induced by inflammatory cytokines such as IL1β.

SFN has in fact been shown to decrease production of PGE2 in several cell culture models [24], [35], [36]. Here, we investigate the mechanism of SFN suppression of PGE2 in A549 human lung cancer cells.

**Figure 1:**
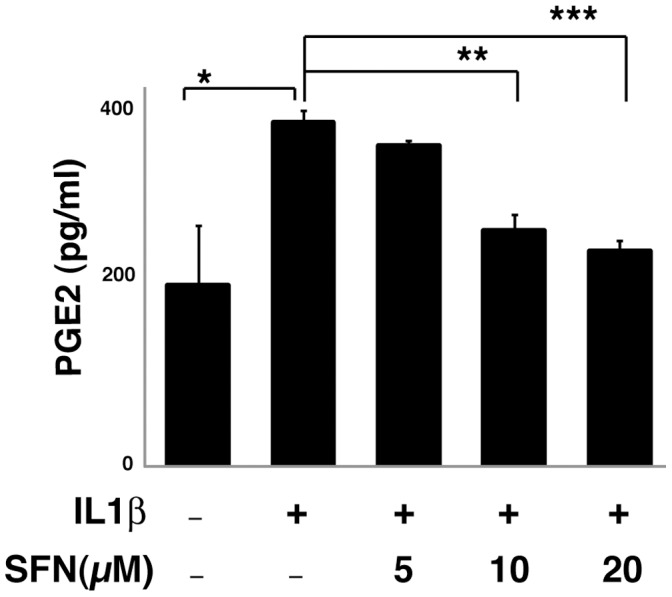
SFN dose dependently inhibits IL1β induced PGE2 production. A549 cells were pretreated for 30 minutes in the presence or absence of SFN and then cultured with or without 1 ng/ml IL1β for 24 hours. PGE2 in the media was measured using EIA assay. *, p<0.05, **, p<0.01, ***, p<0.001 (Student's t test). Data are presented as mean ± SEM (n = 3).

## Methods

### Reagents and Antibodies

Human recombinant interleukin-1β (IL-1β) was purchased from PeproTech Inc (Rocky Hill, NJ). SFN was purchased from LKT Laboratories, Inc (St. Paul, MN). Dulbecco’s modified Eagle’s medium (DMEM) and Trizol were obtained from Invitrogen (Carlsbad, CA). Fetal bovine serum was purchased from HyClone, Thermo Fisher (Waltham, MA). α-tubulin antibody (T6199), and penicillin/streptomycin were purchased from Sigma-Aldrich (St. Louis, MO). mPGES-1 antibody (#160140), prostaglandin H2 (#17020), monoclonal anti-human COX-2 (#160112), and prostaglandin E2 EIA kit (#514010) were purchased from Cayman Chemical (Ann Arbor, MI). Polyclonal anti-Egr-1 antibody (sc-110) and anti-actin antibody (sc-1615) were obtained from Santa Cruz Biotechnology (Santa Cruz, CA). HIF-1α antibody (MAB1536) was obtained from R&D Systems, Inc. GFP antibody was purchased from Chemicon (#AB3080). Anti-RNA polymerase II antibody (#05-623) was purchased from Upstate/Millipore. All cell lines were obtained from American Type Culture Collection. Non-small cell lung cancer A549 cells and human embryonic kidney 293 cells were maintained in Dulbecco’s modified Eagle’s medium supplemented with 10% FBS and penicillin-streptomycin. Human mammary epithelial adenocarcinoma MCF7 cells were cultured in MEM with 10% FBS, 0.01 mg/ml insulin and penicillin-streptomycin and murine mammary epithelial 4T1 cells in RPMI with 10% FBS and penicillin-streptomycin.

**Figure 2:**
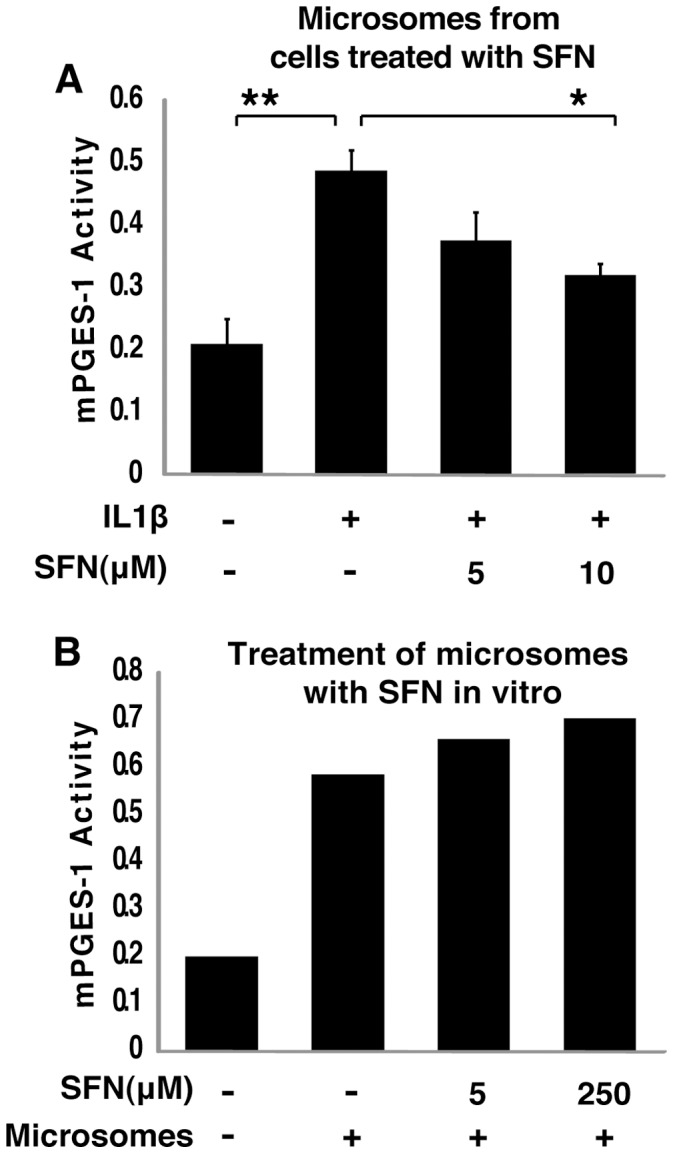
SFN reduces mPGES-1 activity in treated cells but does not inhibit activity in purified microsomes. A, SFN treatment of intact cells inhibited mPGES-1 activity, A549 cells were pretreated with or without different concentrations of SFN for 30 minutes and then 1 ng/ml IL1b was added for another 24 hours. mPGES-1 activity was assayed as conversion of PGH2 to PGE2 by microsomal fractions in a mass-spectrometry assay with PGE2(D4) as internal standard. The chromatographic peak height under the m/z = 351.2 ion elution was normalized by internal standard (PGE2d4) peak height under the m/z = 355.2. *, P<0.05, **, P<0.01 vs. IL1b treated samples (mean ± SEM; n = 3). B, Addition of SFN to purified microsomes in vitro did not inhibit mPGES-1 activity. Microsomal fractions of A549 cells were collected and treated with or without different concentrations of SFN for 2 hours and then mPGES-1 activity was assayed as A.

### Immunoassays for PGE2

PGE2 was measured by prostaglandin E2 EIA kit per manufacturer’s instructions. For measurement of PGE2 concentration in cell culture medium, A549 (2 × 10^5^) cells were cultured with IL1β and SFN for 24 hours. For measurement of PGE2 concentration from tissue samples, the samples were processed using a published protocol [37]. PGE2 concentrations were normalized to tumor weight.

**Figure 3:**
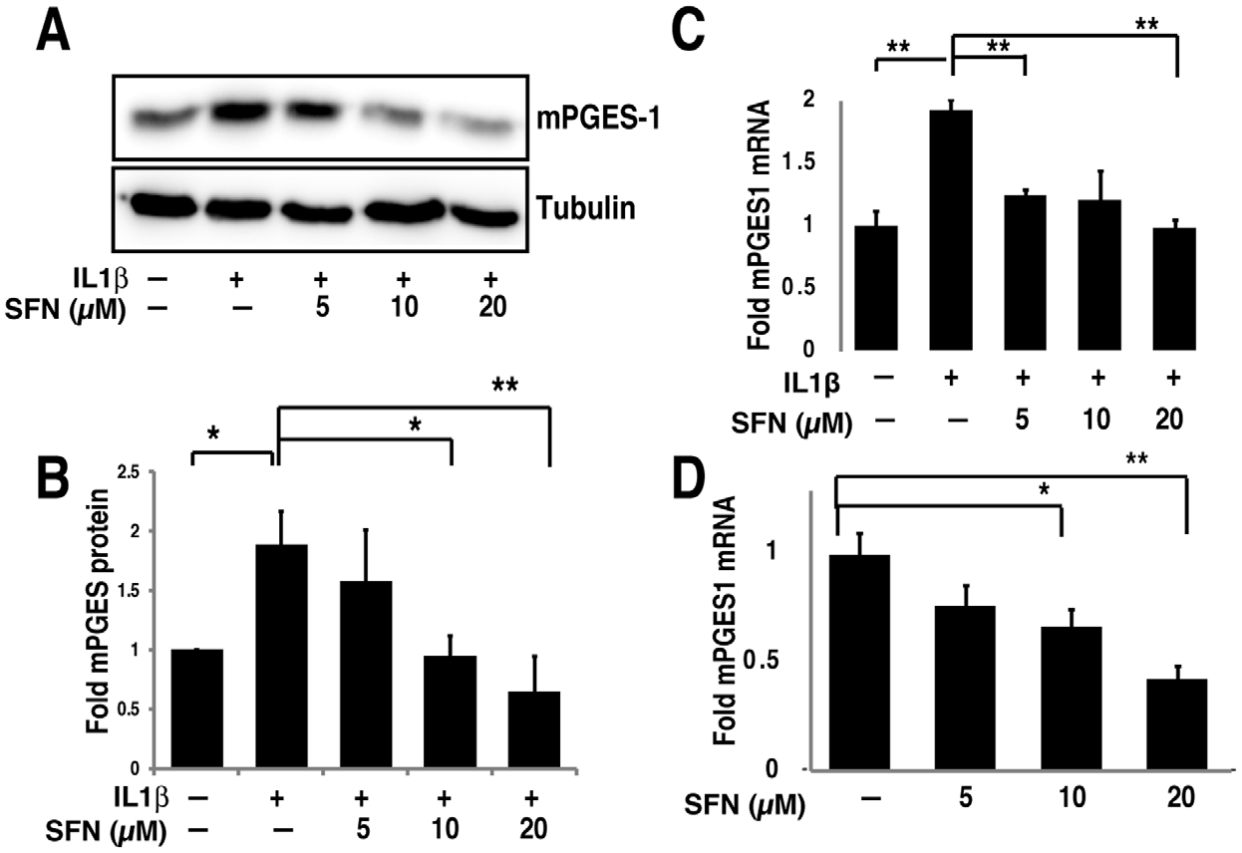
SFN suppresses mPGES-1 expression by inhibiting mRNA transcription. A&B. A549 cells were pre-treated with or without different concentrations of SFN for 30 minutes. IL1β (1 ng/ml) was then added and cells were cultured for another 24 hours. Total cell lysates were subjected to immunoblot with mPGES-1 antibody. Immunoblot shown (A) is representative of three experiments, that are quantified in panel B, with results expressed as mean fold change ± SEM (n = 3). C&D, A549 cells were pretreated with or without SFN for 30 minutes. IL1β (1 ng/ml) was then added (C) or not (D) and cells were cultured for another 4 hours. Total RNA was analyzed by quantitative RT-PCR. The results are expressed as mean fold change ± SEM (n = 3). *, P<0.05, **, P<0.01, ***, P<0.001 (Student's t test).

### Transient Transfection

Transient transfection experiments were performed using Lipofectamine 2000 reagent (Invitrogen) according to the manufacturer’s protocol. Cultured cells were seeded 24 hours prior to transfection at a density of 2 × 10^5^ cells/well and transiently transfected with or without HIF-1α (in pCMV, purchased from Origene Technologies, Inc., MD) and pCMV GFP expression vectors. The total amount of transfected DNA was kept constant using empty vector. After transfection, the cells were treated with increasing concentrations of SFN or vehicle for 40 hours. Total cell extracts were prepared for immunoblot analysis.

**Figure 4:**
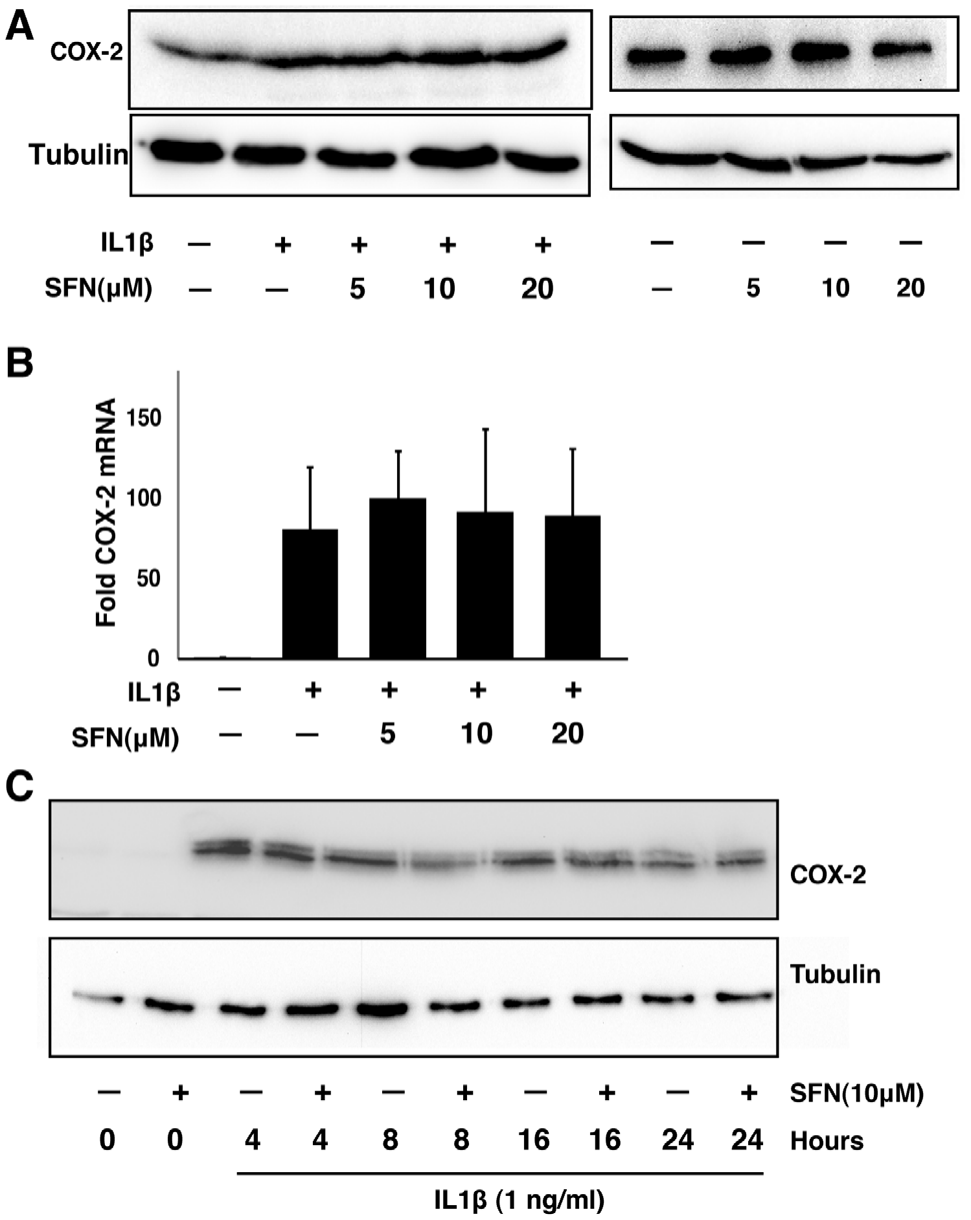
SFN does not inhibit COX-2 expression in A549 cells. A, A549 cells were pre-treated with or without different concentrations of SFN for 30 minutes. IL1β (1 ng/ml) was then added (left) or not (right) and cells were cultured for another 24 hours. Total cell lysates were subjected to immunoblot analysis. B, A549 cells were pretreated with or without SFN for 30 minutes. IL1β (1 ng/ml) was then added and cells were cultured for another 4 hours. Total RNA was analyzed by RT-PCR. The results are expressed as fold change relative to untreated cells (mean ± SEM; n = 3). C, A549 cells were treated with 1 ng/ml IL1β either with or without 10 µM SFN for different times. Total cell lysates were subjected to immunoblot analysis.

### Cell Treatments and Immunoblot Analysis

Cells (2 × 10^5^) were cultured to 80–90% confluence, and then exposed to SFN for indicated times. Whole cell lysates were prepared with DTT/SDS lysis buffer, resolved on SDS-PAGE and transferred to a PVDF membrane (Immobilon, Millipore). Proteins were detected by immunoblotting using antibodies against mPGES-1, COX-2 and HIF-1α followed by incubation with horseradish peroxidase-conjugated secondary antibody. The signals were visualized by chemiluminescence detection system (Alpha Innotech). α-Tubulin served as a loading control.

**Figure 5:**
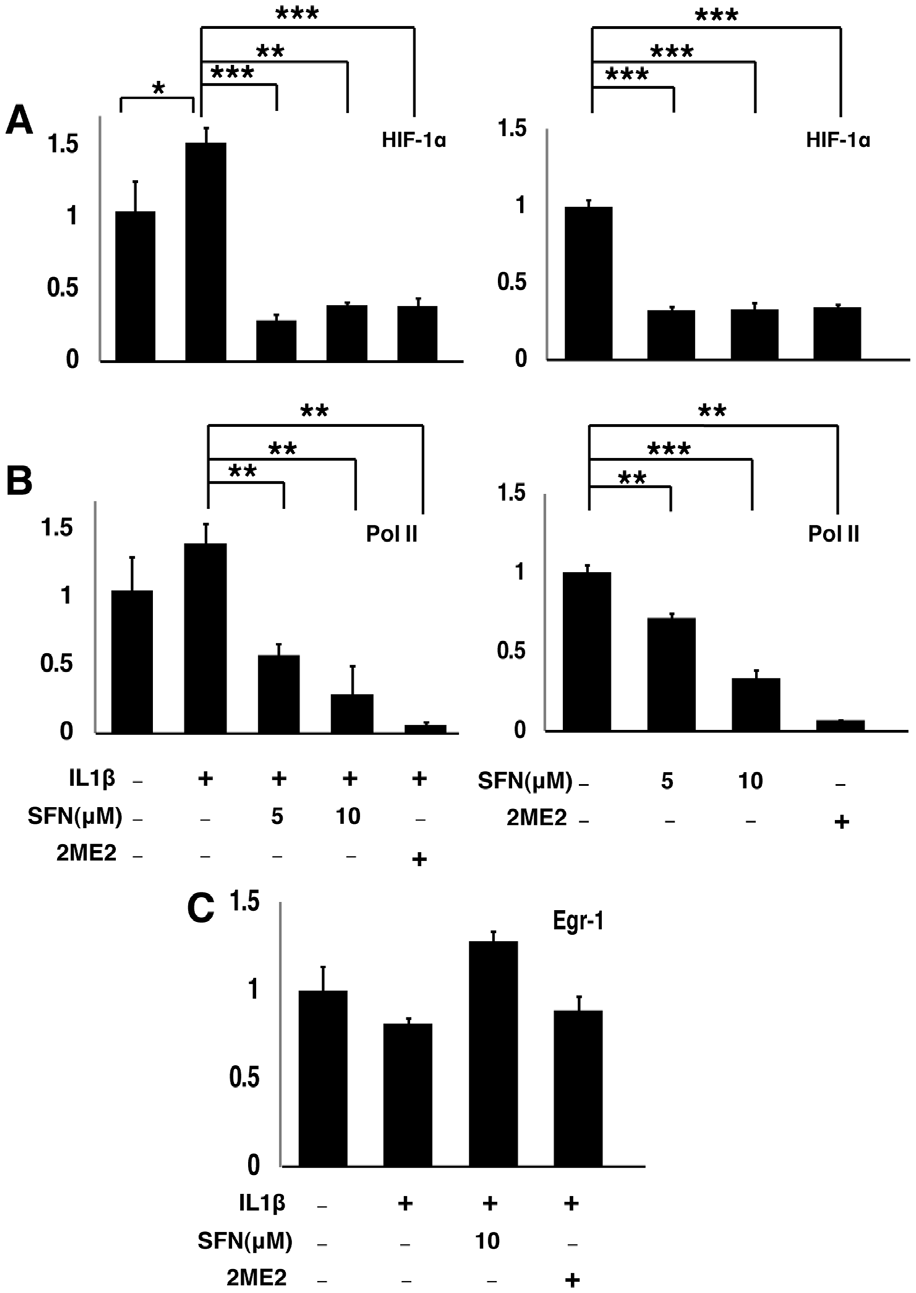
SFN dose dependently inhibits HIF-1α and Pol II occupancy at the mPGES-1 promoter. A549 cells were pretreated with or without SFN and HIF-1α inhibitor 2-Methoxyestradiol (2ME2, 0.2 mM) for 30 minutes. IL1b (1 ng/ml) was either added (left panels) or not (right panels) and cells were cultured for another 4 hours. ChIP assays, coupled with quantitative real time PCR, were performed using HIF-1α (A), Pol II (B) or Egr-1(C) antibodies. The results are expressed as fold change of protein binding to the mPGES-1 promoter relative to untreated cells. (mean ± SEM; n = 3) *, P<0.05, **, P<0.01, ***, P<0.001 (Student's t test).

### Assessment of mRNA Expression

Total RNA was isolated from A549 cells (2 × 10^5^) using Trizol (Invitrogen, CA). First strand cDNA was synthesized using iScript cDNA Synthesis Kit (Bio-rad, Hercules, CA) according to the manufacturer’s instructions. Real time PCR was performed using iQ SYBR Green Real-Time PCR Supermix (Bio-Rad, Hercules, CA) with primers for mPGES-1 (forward 5′-CACAGCCTGGTGATGAGC-3′ and reverse 5′-CCGCTTCCCAGAGGATCT-3′); COX-2 (forward 5′-TATACTAGAGCCCTTCCTCCTGTGCC-3′ and reverse 5′-ACATCGCATACTCTGTTGTGTTCCC-3′); Glut1 (forward 5′-GATTGGCTCCTTCTCTGTGG-3′ and reverse 5′- TCAAAGGACTTGCCCAGTTT); PGK1 (forward 5′-ATGGATGAGGTGGTGAAAGC-3′ and reverse 5′- CAGTGCTCACATGGCTGACT); and β-actin (forward 5′-CCCAGAGCAAGAGAGGTATC-3′ and reverse 5′-AGAGCATAGCCCTCGTAGAT-3′), all synthesized by Invitrogen. The mRNA levels were normalized using β-actin as internal control. The conditions for all reactions were as follows: denaturation at 94°C for 2 minutes and 25 cycles of reactions of denaturation at 98°C for 10 seconds, annealing at 59°C for 30 seconds, and elongation at 72°C for 45 seconds.

**Figure 6:**
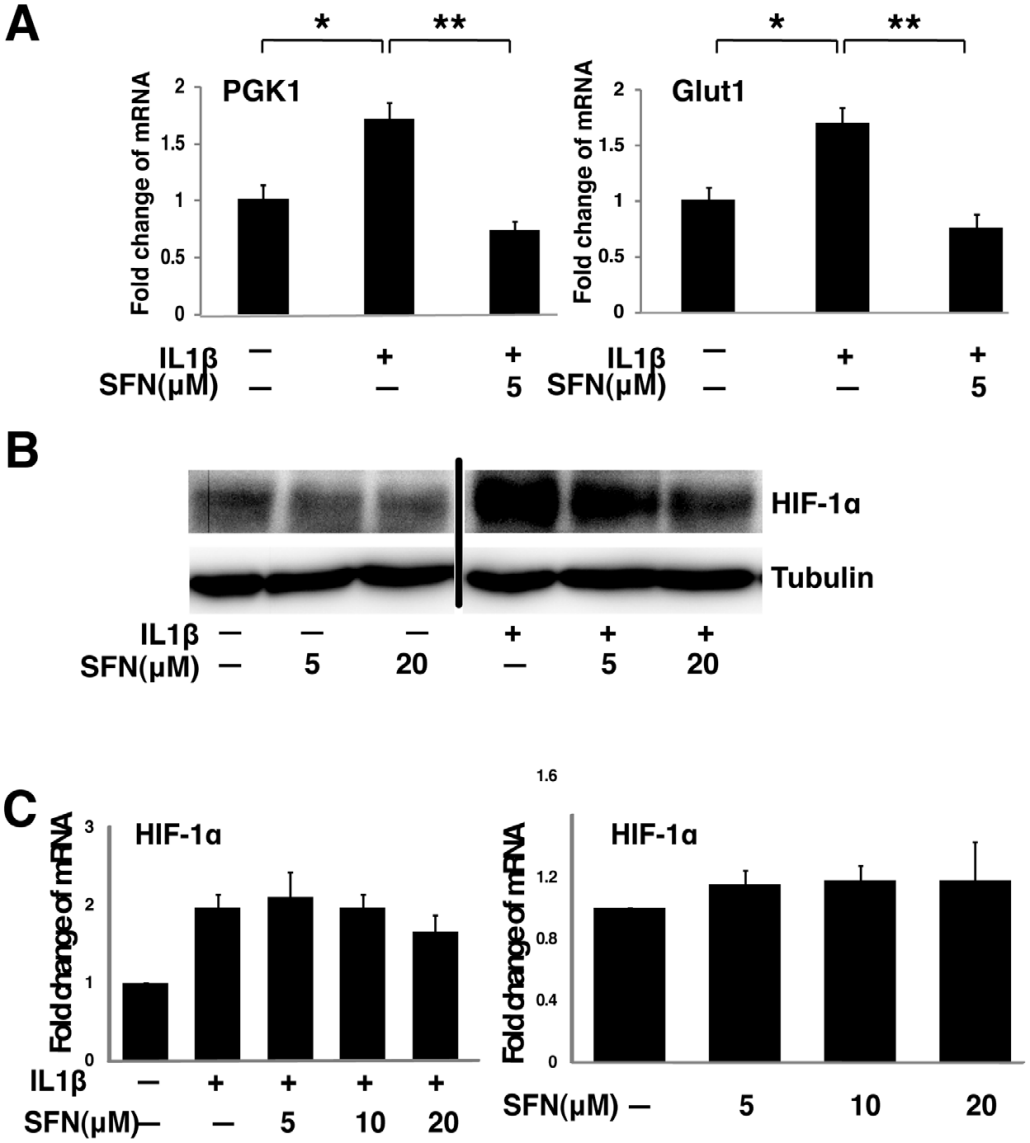
SFN suppresses the HIF-1–mPGES –PGE2 axis by control of HIF-1α protein expression without altering mRNA levels. A, A549 cells were pretreated with or without SFN for 30 minutes. IL1β (1 ng/ml) was then added and cells were cultured for another 4 hours. Total RNA was analyzed by quantitative RT-PCR. The results are expressed as fold change relative to untreated cells (mean ± SEM; n = 3). B&C, A549 cells were pretreated with or without SFN for 30 minutes. IL1β (1 ng/ml) was then added or not and cells were cultured for another 4 hours. Total cell lysates were subjected to immunoblot analysis (panel B) and total RNA was analyzed by quantitative RT-PCR (panel C).

### Chromatin Immunoprecipitation (ChIP) and Real Time Quantitative RT-PCR Analysis

ChIP assay was performed using the ChIP assay kit from Upstate/Millipore. After treatment, A549 cells (1 × 10^6^) were cross-linked with 1% formaldehyde for 10 minutes at 37°C. The fixed cells were washed twice with ice-cold phosphate-buffered saline supplemented with protease inhibitors (1 mM phenylmethylsulfonyl fluoride (PMSF), 1 µg/ml aprotinin and 1 µg/ml pepstatin A) and then lysed for 10 minutes on ice with 200 µl of SDS lysis buffer (1% SDS, 10 mM EDTA, 50 mM Tris, pH 8.1) containing protease inhibitors. The chromatin samples were sonicated to reduce DNA length to 500–700 bp. The supernatant was diluted 1∶10 in ChIP dilution buffer (0.01% SDS, 1.1% Triton X-100, 1.2 mM EDTA, 16.7 mM Tris-HCl, pH 8.1, 167 mM NaCl) plus protease inhibitor and 20 µl of the chromatin samples were saved as the input DNA material. The chromatin samples were pre-cleared with Protein A agarose/salmon sperm DNA 50% slurry (Millipore) for 30 minutes. The samples were then incubated with mixing overnight at 4°C with antibodies specific for either HIF-1α, RNA pol II, Egr-1 or absence of antibody as negative control. Immune complexes were recovered by addition of salmon sperm DNA/protein A-agarose slurry for 1 hour. The immunoprecipitates were sequentially washed twice with low salt, high salt, LiCl and TE buffer and eluted twice with 250 µl elution buffer (1% SDS, 0.1 M NaHCO3) for 15 minutes. The eluted material and the DNA input samples were incubated in 200 mM NaCl for 4 hours at 65°C to reverse cross-linking and then treated with proteinase K for 1 hour at 45°C. Finally DNA was recovered by phenol/chloroform extraction and ethanol precipitation and subjected to real time PCR analysis using primers directed against the human mPGES-1 promoter (sense 5′-CCCGGAGACTCTCTGCTTC-3′ and antisense 5′-TCAACTGTGGGTGTGATCAGC-3′).

**Figure 7:**
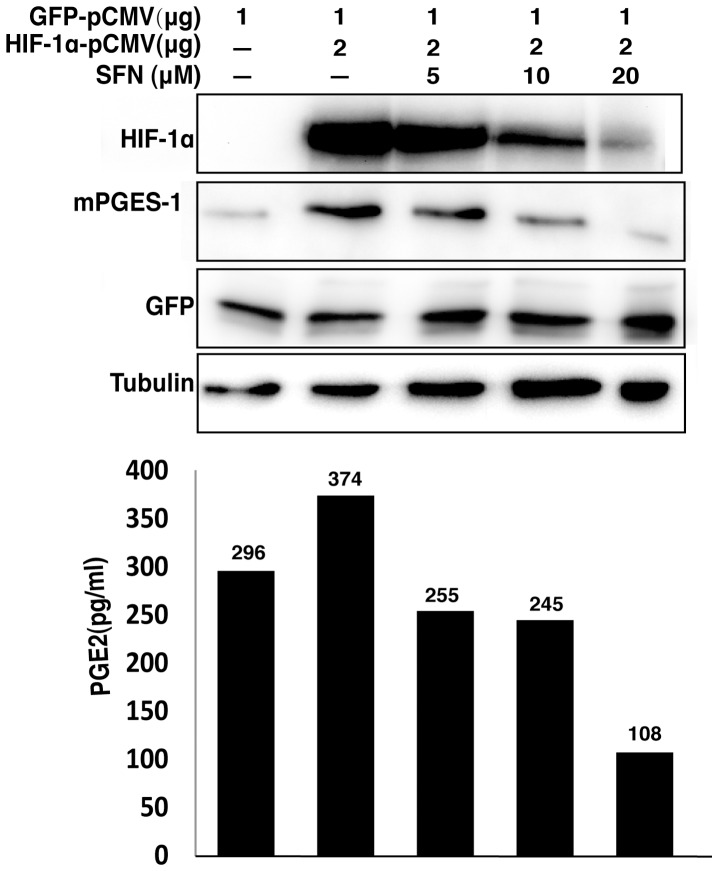
SFN suppresses mPGES expression and PGE2 production driven by overexpression of HIF1α. A549 cells were transfected with indicated amounts of HIF-1α and GFP expression vectors. The total amount of transfected DNA (4 µg) was kept constant by addition of the empty vector. The cells were then left untreated or treated with increasing concentrations of SFN for 40 hours. The total cell extracts were prepared for immunoblot analysis to evaluate protein levels. The cell culture media were used for measuring PGE2 concentration by EIA assay.

### Cellular Fractionation

The method was derived from a published protocol [38]. Cells (1 × 10^6^) were collected by scraping and homogenized (Dounce glass homogenizer) in 500 µl of 0.1 M K-PO4 buffer, pH 7.5; 0.5 M sucrose; 1 mM reduced glutathione (GSH) with complete protease inhibitor cocktail (2.5 µg/ml aprotinin and leupeptin). The homogenates were centrifuged at 12,000×g, 4°C for 15 minutes, and the supernatant was then further fractionated at 100,000×g (Airfuge® Air-Driven Ultracentrifuge at 30 psi), 4°C for 2 hours. Pellets (microsomal fraction) were resuspended in 400 µl of 0.1 M K-PO4, pH 7.5; 2.5 mM GSH; and 10% (vol/vol) glycerol. The microsomal protein concentration was quantified by Bradford colorimetric assay.

**Figure 8:**
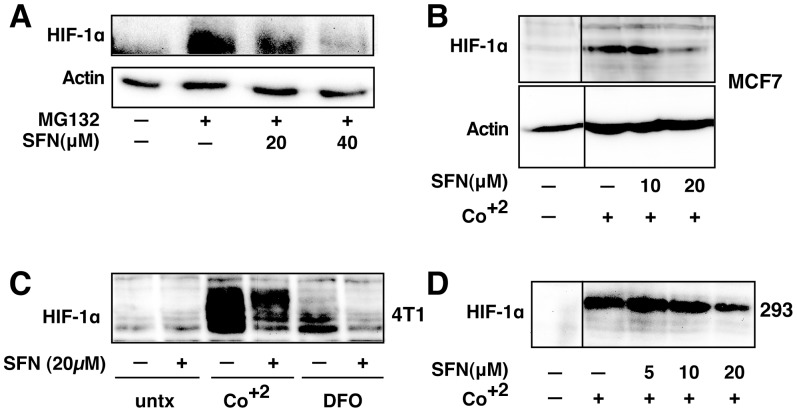
SFN inhibits HIF-1α protein accumulation. Cell lines were treated as described below, and total cell lysates subjected to immunoblot analysis. A, A549 cells were pretreated with or without SFN and MG132 (20 µM) for 1 hour. B, MCF7 cells were pretreated for 1 hour with indicated concentration of SFN, and then treated with 100µM cobalt chloride (Co^+2^) for an additional 5.5hours. C, 4T1 cells were pretreated for 20 min with 20 µM SFN, followed by treatment with 250µM cobalt chloride or 200µM desferroxamine as indicated for 4hours. D. 293 cells were pretreated for 20 min with indicated concentrations of SFN, followed by stimulation with 250 µM cobalt chloride for an additional 4 hours.

### mPGES-1 Enzyme Activity Assay

8 µg of microsomal protein was incubated with SFN or solvent control in 50 µl of reaction buffer (100 mM sodium phosphate pH 7.2, containing 2.5 mM GSH, 1 mM EDTA, 5 mM Triton X-100) for 2 hours at 20°C. The enzyme reaction was initiated by adding 40 µM PGH2, and incubated for 30 seconds on ice. Reaction was terminated by adding 0.4 ml of 20 mM FeCl_2_ and 50 mM citric acid, pH 2–3. Unreacted PGH2 was decomposed into 12-hydroxy-heptadecatrienoic acid and malondialdehyde by FeCl_2_. PGE2d4 (deuterated, 0.25 µg) in 50 µl of ethanol (final concentration 10%) was added as internal standard and samples (500 µl) were further processed by solid-phase extraction for mass spectrometry analysis.

**Figure 9:**
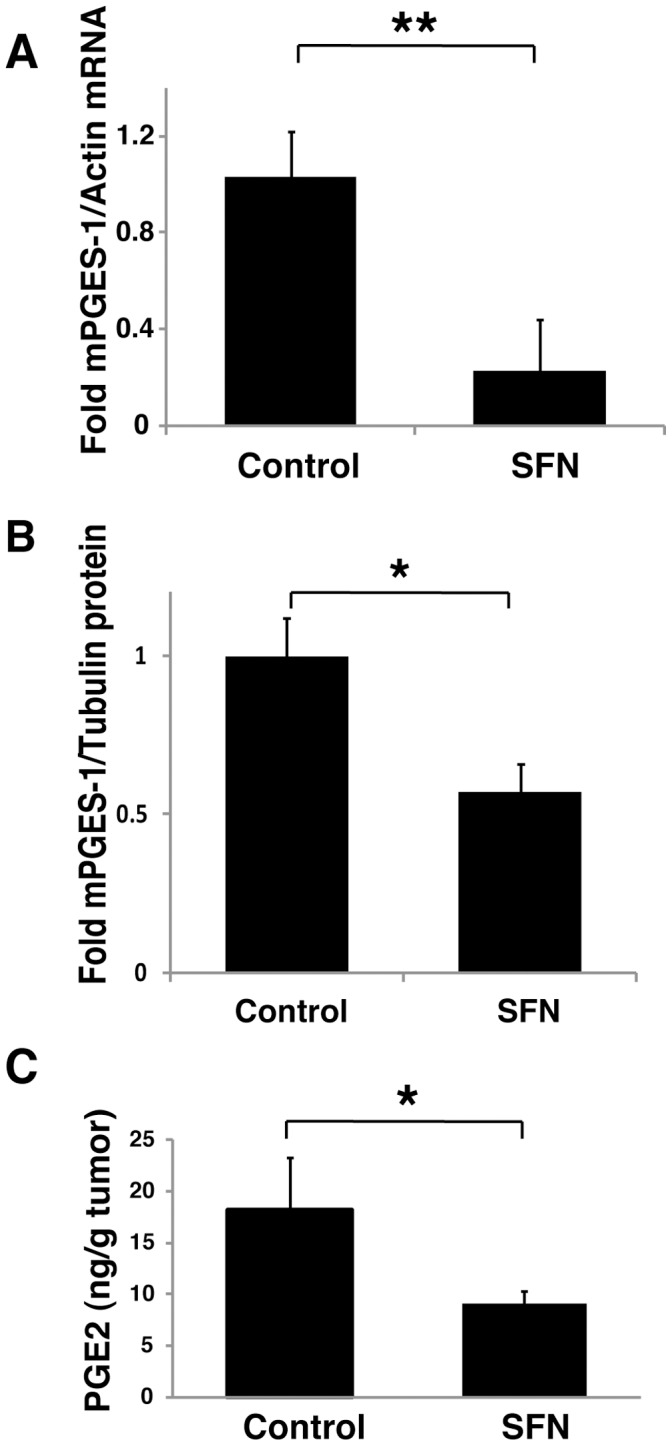
SFN inhibits mPGES-1 expression and PGE2 production in tumors *in vivo*. 2.5 × 10^6^ A549 cells were injected subcutaneously on the flank of athymic nude mice. After 6 weeks, animals were treated with PBS vehicle (control) or 0.5 mg SFN by intraperitoneal injection and sacrificed 24 hours later. Tumor tissue samples were weighed and frozen for subsequent RT-PCR assay (A), immunoblot analysis (B) and PGE2 measurement (C). The results in A and B are expressed as fold changes relative to PBS treated mice (mean ± SEM; n = 4). *, P<0.05, **, P<0.01, (Student's t test). Four mice in each group (PBS vehicle or SFN treated) were used in this experiment.

### Prostaglandin Isolation and Solid-phase Extraction

Prostaglandin isolation was based on previous procedure [39] as optimized for our MS instrument. Prostaglandins in culture media were extracted using Strata® × SPE columns (Phenomenex, Torrance, CA). Columns were washed with 2 ml of MeOH followed by 2 ml of distilled water. After applying 1 ml of each sample including internal standard, PGE2d4, the columns were washed with 1 ml of 10% MeOH, and the PGs were then eluted with 1 ml of MeOH. The eluant was dried under vacuum and redissolved in 100 µl of 0.5 M acetic acid and 0.1 µl of samples were loaded into a Finnigan LTQ ion trap mass spectrometer (Thermo Electron, San Jose, CA).

**Figure 10:**
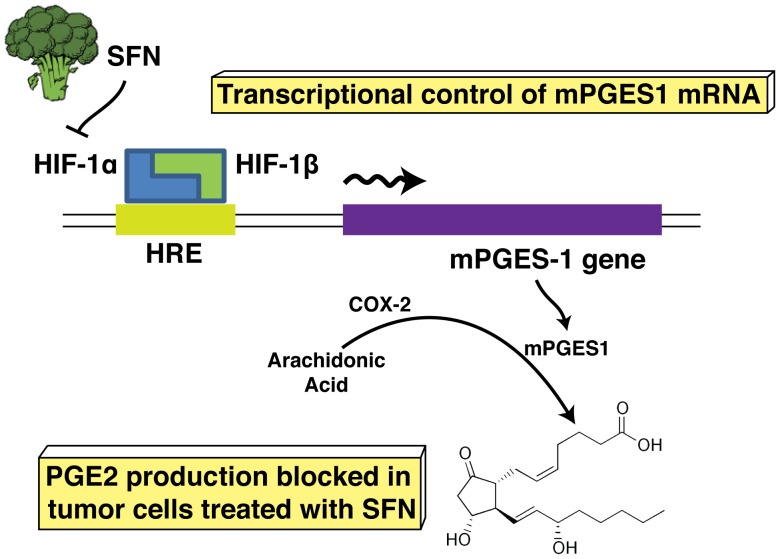
A model for the role of SFN in the regulation of PGE2 production. SFN inhibits HIF-1α protein abundance, which leads to a reduction of HIF-1α on the promoter of mPGES-1 gene. This results in inhibition of transcription of mPGES-1 and suppression of PGE2 production.

### HPLC-MS-MS

The extracted prostaglandins were pressure-loaded onto a 360 µm o.d.×75 µm i.d. microcapillary fused silica column (Polymicro Technologies, Phoenix, AZ) that was packed in-house with C18 resin and then connected a 10–15 µm tip suitable for electrospray. The samples were gradient eluted by using a model 1100 series HPLC solvent delivery system (Agilent, Palo Alto, CA) split to nano-flow. Using a Thermo LTQ-XL ion trap mass spectrometer (Thermo, San Jose, CA), electrospray ionization was performed in the negative ion mode with a flow rate of 100 nl/min (Solvent A, 0.5 M acetic acid; solvent B, 0.5 M acetic acid/80% acetonitrile/20% water). The HPLC gradient used was 0–50% solvent B for 3 minutes, 50–100% solvent B for 15 minutes, 100% solvent B for 17 minutes, and then, solvent B was dropped to 0% for 5 minutes and held for 20 minutes. The mass spectrometer was operated in a data dependent mode where an initial full MS1 scan recorded the mass-to-charge (m/z) ratios of lipid ions over the mass range 300–2000 m/z. The chromatographic peak height for the -1 charge state of PGE2 (m/z = 351.2) was normalized by the peak height for the -1 charge state of the PGE2d4 internal standard (m/z = 355.2). The identity of these PGE2 and PGE2d4 peaks was verified by comparing their MS2 spectra with those obtained for PGE2 and PGE2d4 standards (Cayman Chemical).

### Animal Model

Animals were maintained in the UVA animal facility under the auspices of an IACUC approved protocol. For the xenograft model, 2.5 × 10^6^ A549 cells were injected subcutaneously on the flank of athymic nude mice. After 6 weeks, animals were treated with PBS vehicle or 0.5 mg SFN by intraperitoneal injection and sacrificed 24 hours later. Tumor tissue samples were weighed and frozen for subsequent RT-PCR assay and PGE2 measurement.

### Statistical Analysis

Statistical analysis was performed using Student’s t-test. In all figures, P values are indicated as *, P<0.05, **, P<0.01, ***, P<0.001.

## Results

### SFN Blocks Production of PGE2 in Human Cancer Cells

Because of the importance of PGE2 to human cancers and the role of SFN in cancer chemoprevention, we examined the effect of SFN on PGE2 production in a human lung carcinoma cell line (A549). We treated A549 cells for 24 hours with IL1β, in the presence or absence of SFN. PGE2 production, measured using an enzyme-linked immunoassay, was increased nearly two fold after IL1β stimulation (Figure 1) and SFN dose-dependently reduced PGE2 production. Thus SFN inhibited PGE2 production in this A549 cancer cell line.

### SFN Suppresses mPGES-1 Activity through an Indirect Mechanism

To determine the mechanism by which SFN blocks production of PGE2 in human cancer cells, we developed an in vitro assay for PGE2 synthesis from its precursor PGH2. The major inflammation induced biosynthetic enzyme that participates in this conversion is microsomal prostaglandin E synthase 1 (mPGES-1) [40]. We measured mPGES-1 activity in A549 cells by isolating microsomes from cells that were untreated, treated with IL1β alone, or treated with a combination of IL1β and SFN, and then used these isolated microsomes to convert PGH2 to PGE2 *in vitro*. PGE2 production was quantified using liquid chromatography-mass spectrometry (LC-MS). The mPGES-1 activity in purified microsomes derived from SFN treated cells was decreased (Figure 2, panel A).

We have previously shown that SFN covalently modifies and inhibits the enzyme activity of Macrophage Migration Inhibitory Factor (MIF) by binding to its amino terminal proline residue, and that this covalent modification of MIF is easily replicated by treatment with SFN in vitro [25]. We noted that, while it otherwise bears little sequence similarity to MIF, mPGES-1 also has an amino terminal proline. We considered whether SFN might inhibit mPGES-1 through covalent modification and enzymatic silencing, similar to the mechanism through which SFN silences MIF. However, SFN added directly to microsomal preparations from untreated A549 cells did not block mPGES-1 activity (Figure 2, panel B), demonstrating that direct covalent modification, or other forms of direct enzyme activity suppression, is not the mechanism through which sulforaphane reduces mPGES-1 activity in cells.

### SFN Reduces Expression of mPGES-1 Protein and mRNA

To determine the mechanism of SFN suppression of mPGES-1 activity, we examined expression of both mPGES-1 protein and mRNA. We stimulated A549 cells with IL1β in the absence or presence of SFN for 24 hours and then evaluated mPGES-1 protein expression by immunoblotting. We demonstrated that SFN inhibited IL1β-induced mPGES-1 expression in a dose-dependent manner (Figure 3A; quantified over several experiments in Figure 3B). Coincident with the reduced mPGES-1 protein expression, we also noted a dose dependent decrease in mPGES-1 mRNA levels following SFN treatment, measured by quantitative PCR, both in the presence (Figure 3C) and absence (Figure 3D) of IL1β. Thus SFN controls mPGES-1 expression, and thereby PGE2 production, at the level of mPGES-1 mRNA abundance.

Because of the importance of COX-2 in controlling prostaglandin expression, we also examined COX-2 expression in the same samples used in Figure 3A and C, by using both immunoblotting (Figure 4A) and quantitative PCR (Figure 4B). Neither COX-2 protein nor mRNA levels were altered by SFN at the time point when mPGES-1 was inhibited. We also examined COX-2 in time course experiments, and found that while SFN transiently and modestly reduced COX-2 protein expression after 8 hours of treatment, expression of COX-2 was restored at later time points (Figure 4C). Together, these data suggest that reduction of mPGES-1 rather than COX-2 is the cause for the observed inhibition of PGE2 production in A549 cells.

### SFN Inhibits the Presence of HIF-1α at the mPGES-1 Promoter

Transcription of mPGES-1 is regulated by several transcription factors, including early growth response gene 1 (Egr-1) [41], [42] and HIF-1 [43]. Both factors regulate a number of genes involved in inflammation and cancers [44]–[46]. Egr-1 binds to the GC box (5′-GCG[G/T]GGGCG-3′) [47], [48] and HIF-1α binds to HIF-responsive elements ([A/G]CGT[G/C]C) [43]. The mPGES-1 promoter contains two known GC boxes and three potential HIF-responsive elements. A dominant-negative HIF-1α lacking DNA binding and transactivation domains reduces mPGES-1 mRNA and protein levels [43], confirming that HIF-1α regulates mPGES-1 transcription either directly or indirectly.

We performed chromatin immunoprecipitation (ChIP) assays using HIF-1α and EGR-1 specific antibodies to evaluate the involvement of these factors in SFN-regulated mPGES-1 transcription in A549 cells. As shown in Figure 5A (left panel), treatment with IL1β increased the abundance of HIF-1α associated with the proximal region of the mPGES-1 promoter. Pretreatment with SFN strongly reduced the promoter occupancy by HIF1α. As a positive control, HIF-1α association with the mPGES-1 promoter was also inhibited by 2-methoxyestradiol (2ME2), a characterized HIF-1 inhibitor [49]. In contrast, the promoter occupancy by EGR-1 was not affected by SFN or 2ME2 (Figure 5C). Similar results were also seen in the absence of IL1β treatment (Fig. 5A, right panel). Thus, SFN inhibits HIF-1α occupancy of the mPGES-1 promoter in A549 cells, but has no effect on Egr-1 binding.

Active transcription is accompanied by RNA pol II localization to an active promoter. We performed ChIP assays with RNA pol II antibody to determine whether treatment of A549 cells with SFN affects RNA pol II loading on the mPGES-1 proximal promoter. As shown in Figure 5B, the prevalence of RNA pol II at the mPGES-1 proximal promoter was dose dependently inhibited by SFN both in the presence (left panel) and absence (right panel) of IL1β induction. Together, these results suggest that SFN specifically inhibits mPGES-1 transcription by reducing HIF-1α occupancy at the mPGES-1 promoter.

### SFN Controls HIF-1 Function by Inhibiting HIF-1α Protein Levels

Similar to the effects of SFN on mPGES-1 transcription, the transcription of two known HIF-1α downstream target genes, Glut1 and PGK1, was also suppressed by SFN (Figure 6A). This suggests that SFN inhibits expression of other HIF-1 dependent genes. Since HIF-1 activity is controlled by several mechanisms that regulate the expression levels of the HIF-1α protein, we tested if HIF-1α protein level is regulated by SFN. We treated A549 cells with IL1β and SFN and examined HIF-1α expression by immunoblot. We found that HIF-1α protein was induced by IL1β and HIF-1α protein levels were inhibited by SFN in a dose-dependent manner in both the presence and absence of IL1β treatment. Thus, SFN reduces HIF-1α protein abundance (Figure 6B). This inhibition of HIF-1α abundance was not accompanied by suppression of HIF-1α mRNA levels (Figure 6C), suggesting that SFN suppresses HIF-1α at a posttranscriptional level.

When HIF-1α expression was increased in A549 cells by transfection with a CMV-based expression vector encoding a full length HIF-1α cDNA, HIF-1α expression was dose-dependently inhibited by SFN (Figure 7). Co-expression of GFP from a vector with an identical promoter was equivalent in SFN-treated and control cells, demonstrating that the CMV promoter was not regulated by SFN. Accompanying this forced expression of HIF-1α, we found increased mPGES-1 expression in transfected cells and increased PGE2 production in the cell culture media (Figure 7). Coordinately with the reduction in HIF-1α, SFN treatment of the HIF-1α transfected cells reduced mPGES-1 protein levels and PGE2 concentrations. These data strongly support the model that SFN inhibits mPGES-1 expression and PGE2 production through suppression of HIF-1α.

### SFN Suppresses HIF-1α Protein Induction by Other Stimuli

To determine whether the effects of SFN on HIF-1α are generalizable, we examined HIF-1α protein abundance after different stimuli in several cell lines. Treatment of A549 cells with the proteasome inhibitor, MG132, resulted in a dramatic increase in HIF-1α that was dose responsively inhibited by SFN (Figure 8A). Likewise, HIF-1α induction by cobalt treatment, a hypoxia mimic, was inhibited by SFN in MCF7 (Figure 8B), 4T1 (Figure 8C) and 293 cells (Figure 8D). Finally, HIF-1α induction by a second hypoxia mimic, desferroxamine, was also blocked by SFN in 4T1 cells. Taken together, these data suggest that SFN suppresses HIF1 protein abundance induced by several independent stimuli in a number of different cell lines.

### SFN is Effective at Blocking the mPGES-1/PGE2 Axis in vivo

Finally, we examined the effects of SFN on mPGES-1 and PGE2 in vivo, using immunodeficient mice bearing tumors derived from A549 cells. Microsomal PGES-1 mRNA (Figure 9A), mPGES-1 protein (Figure 9B) and PGE2 levels (Figure 9C) were significantly reduced in the A549 tumors from animals twenty four hours after treatment with a single dose of SFN. Taken together, our results indicate that SFN is effective at blocking the mPGES-1/PGE2 axis both in vivo and in vitro.

## Discussion

To summarize our observations, we propose a model for inhibition of HIF-1α, mPGES-1 and PGE2 in human A549 cells (Figure 10). These results present new information supporting the role of SFN in controlling PGE2 expression in cancer. While inhibitory effects of SFN on COX-2 have been reported previously [50], we did not observe sustained inhibition of COX-2 in response to SFN. Instead, we identified mPGES-1 transcription as the proximate inhibitory target of SFN in control of PGE2 production in A549 cells.

PGE2 is increasingly recognized as a regulator of cancer. The importance of PGE2 is underscored by the frequent overexpression of COX-2 [51] and the frequency with which the PGE2 metabolizing enzyme 15 hydroxy-prostaglandin dehydrogenase (15-PGDH) is transcriptionally silenced in cancers [52]. The mechanism through which PGE2 promotes cancer is not clear, but we note that PGE2 has been described as important for attraction or commitment of tumor-supporting myeloid derived suppressor cells (MDSCs) [34]. Thus, SFN may suppress the formation of the tumor-supporting microenvironment through inhibition of PGE2 production. This hypothesis is under study in our laboratory currently. If true, this mechanism is entirely distinct from the prevailing hypothesis that chemopreventives prevent carcinogenesis through detoxification of mutagens, as discussed above. Instead, SFN and potentially other cancer prevention compounds might serve (through blockade of PGE2) to prevent formation of a hospitable environment for both nascent and metastatic tumors. Thus, cancer preventives may provide therapeutic benefit for cancer recurrence or metastasis.

COX inhibitors, including aspirin and other non-steroidal anti-inflammatory drugs (NSAIDs) effectively reduce PGE2 production. However, NSAIDs are associated with negative health effects, largely due to side effects such as anticoagulation or unintended reduction of other lipid mediators. Selective COX-2 inhibitors have, or had, the potential to avoid these negative consequences of NSAIDs. However, long-term use of selective COX-2 inhibitors leads to high incidence of sudden myocardial infarction and thrombosis [53]. Thus, suppression of mPGES-1 represents an attractive alternative approach to reducing PGE2 in cancers.

PGE2 is also well studied in non-cancer pathologies. It is a central mediator of inflammation, and may contribute to the destructive pathology associated with inflammatory bowel disease, multiple sclerosis, and other inflammatory diseases. PGE2 also inhibits smooth muscle contraction, and over-responsiveness to PGE2 is involved in failure to close the ductus arteriosus in neonates [54] and in relaxation or “ripening” of the uterine cervix during childbirth [55]. Increased mPGES-1 expression and PGE2 production levels are also associated with inflammatory diseases such as atherosclerosis [56] and arthritis [57]. Thus, natural products containing SFN, or pharmacologic homologues might prove useful in clinical situations ranging from blocking inflammatory disease, to prevention of premature labor, and could inhibit PGE2 synthesis with fewer side effects than the NSAIDs.

Most work on transcriptional regulation of mPGES-1 has focused on regulation by the EGR-1 transcription factor. However, Lee et al. identified three potential HIF-1 responsive element (HRE) sites within 5kb of the predicted transcription start site of the mPGES-1 gene and showed that hypoxia induces mPGES-1 in a HIF-1α dependent manner in esophageal epithelial cells [43]. We provide both positive and negative evidence for HIF-1α being the relevant target of SFN; overexpression of HIF-1α induces mPGES-1 and PGE2 production and SFN reduces HIF-1α occupancy of the mPGES-1 promoter. Therefore, our studies confirm the role of HIF-1α in regulating mPGES-1 transcription and extend upon these studies by identifying a novel mechanism through which SFN controls PGE2 production.

IL1β is a critical cytokine involved in inflammatory processes, including those within the tumor microenvironment [31]. Consistent with our observations, IL1β has been well characterized as an activator of HIF-1α [32], [33]. In human gingival and synovial fibroblasts, IL-1β increases HIF-1α mRNA, as well as increasing the binding of the heterodimer HIF-1 to HIF-1 binding site [58]. In human hepatoma cells, IL-1β also stimulates DNA binding and the protein accumulation of HIF-1α [32], indicating the close relationship between IL-1β and HIF-1α activity. Taken together, this suggests that IL1β induction of HIF-1α may play a significant role in the regulation of gene expression at sites of inflammation, including early in tumorigenesis. While we focus on IL1β as a physiologically relevant inducer of HIF-1α, we also demonstrate that SFN has a similar impact on HIF-1α induced by several other stimuli that function by distinct mechanisms, suggesting that SFN may be more generally useful for inhibiting this critical pathway.

The regulation of HIF-1α dependent PGE2 production by SFN in this study is a new finding. However, connections between hypoxia (presumably involving HIF-1) and mPGES-1 have been seen in chondrocytes [59], human esophageal cells [43] and HT-29 colon cancer cells [60], indicating the close connections between hypoxia and inflammation. Besides inducing PGE2 production, hypoxia is also strongly associated with the progression of malignancy through other pathways, such as angiogenesis and metastasis. VEGF, which plays a key role in angiogenesis progression, is a direct transcriptional target of HIF-1α. On the basis of their importance for cancer cell survival, HIF-1α and VEGF are preferred targets for anti-cancer strategies. Interestingly, SFN reduces angiogenesis [61] and this effect may play an important role in the chemopreventive and anti-cancer effects of SFN.

Our data clearly show that SFN reduces the steady state levels of HIF-1α protein induced by several diverse stimuli, including the physiologically relevant IL1β used throughout the study. Since SFN did not alter the levels of HIF-1α mRNA, this effect is not mediated by inhibition of transcription. HIF-1α is primarily controlled at the level of protein stability. Specifically, synthesized protein is rapidly degraded in following proline hydroxylation-induced ubitquitylation mediated by the E3 ubiquitin ligase, VHL. This degradation is blocked by hypoxia, chemicals such as cobalt and DFO, or by inhibition of the proteasome. Alternatively, HIF-1α protein levels can be regulated through impacts on translation, mediated by an IRES element in the 5′UTR of the HIF-1α mRNA [62]–[63]. Ongoing work in our laboratory is focused on addressing which of these mechanisms is responsive to SFN.

In conclusion, based on these results, we propose a mechanism by which SFN results in reduced transcription of mPGES-1 and, ultimately, inhibition of its product PGE2. Thus, this cancer preventing dietary compound may prove valuable in controlling inflammatory and metabolic processes as well as cancers.
